# Whole-Genome Sequences of Four Indian Isolates of Azospirillum brasilense

**DOI:** 10.1128/MRA.00633-19

**Published:** 2019-08-01

**Authors:** Chhaya Singh, Parul Pandey, Durgesh Narain Singh, Rachna Pandey, Ajit Kumar Shasany, Anil Kumar Tripathi

**Affiliations:** a School of Biotechnology, Institute of Science, Banaras Hindu University, Varanasi, India; b Central Institute of Medicinal and Aromatic Plants (CSIR-CIMAP), Lucknow, India; c Dr. D. Y. Patil Biotechnology and Bioinformatics Institute, Dr. D. Y. Patil Vidyapeeth, Pune, India; University of Southern California

## Abstract

Azospirillum brasilense is used worldwide as a plant growth-promoting inoculant for agricultural crops. To understand how the genomes of Indian strains of *A. brasilense* compare with their South American counterparts, we determined the whole-genome sequences of four strains of *A. brasilense* isolated from the rhizosphere of grasses from India.

## ANNOUNCEMENT

The four strains of Azospirillum brasilense (MTCC4035, MTCC4036, MTCC4038, and MTCC4039) were isolated from the rhizosphere of different grasses and identified on the basis of their phenotypic features and 16S rRNA sequences (1) and deposited in the Microbial Type Culture Collection at the Institute of Microbial Technology in Chandigarh, India. Compared with Brazilian strains of *A. brasilense*, the Indian strains showed considerable phenotypic variability in their osmoregulatory traits, acetylene-reducing ability, exopolysaccharide production, and aggregation ability (1).

Single colonies of each strain were used for obtaining overnight cultures in minimal malate medium. Genomic DNA extraction and quantification were done by using a QIAamp genomic DNA kit and a Qubit fluorometer, respectively. Quality control (QC) was performed on a Pippin prep system (Sage Science, Inc., Beverly, MA, USA). Sheared DNA (5 μg) was treated with SMRTbell template prep kit 1.0 reagents (Pacific Biosciences, Menlo Park, CA, USA) and size selected (10 to 50 kb) on a BluePippin system (Sage Science, Inc.). The sequencing primer V2 was annealed at a final concentration of 0.8333 nM, and the P6 v2 polymerase was bound at 0.500 nM. The libraries were sequenced on a PacBio RS II instrument at a loading concentration (on plate) of 140 pM or 160 pM using the MagBead OneCellPerWell loading protocol, DNA sequencing kit 4.0 v2, SMRT cell v3, and 4-h movies. Assembly was done with an HGAP3 (2–4) workflow with a coverage cutoff of 30× and default settings. The raw subreads were generated from raw.bax.h5 PacBio data files and used for read-error correction and preprocessing for assembly (Celera Assembler [3]). A final polishing was done to reduce the remaining indels and base substitution errors in the draft assembly using BLASR (4) and Quiver. Circular contig confirmation and genome annotation were done using Gepard software (http://cube.univie.ac.at/gepard) and the NCBI Prokaryotic Genome Annotation Pipeline (PGAP) (5) v4.6, respectively. Summary statistics and characteristic features of the whole-genome sequencing, assembly, and annotation of all the four strains are given in Table 1.

**TABLE 1:** Summary statistics of whole-genome sequencing, assembly, and annotation

Characteristic	Data for strain:			
	MTCC4035	MTCC4036	MTCC4038	MTCC4039
No. of raw subreads	110,748	109,352	100,490	94,843
Avg length of reads	13,404	12,899	12,901	12,251
No. of sequenced bases	1,484,488,518	1,410,574,816	1,296,438,219	1,161,946,748
Estimated coverage (×)	203	192	178	160
Total no. of contigs	8	9	6	6
Genome size (bp)	7,928,656	8,121,000	7,134,170	7,196,248
Avg GC content (%)	68.52	68.37	68.31	68.94
Maximum contig length (bp)	3,007,928	3,065,527	3,007,857	2,941,886
*N*_50_ value for assembly (bp)	1,930,550	2,058,394	1,757,525	1,845,095
No. of plasmids	7	8	5	5
Total no. of genes	7,146	7,491	6,518	6,553
No. of coding genes	6,816	6,973	6,180	6,262
No. of RNA genes	118	119	124	118
No. of rRNAs (5S, 16S, 23S)	8, 9, 9	8, 9, 9	9, 10, 10	8, 9, 9
No of tRNAs	88	89	91	88
No. of ncRNAs^*a*^	4	4	4	4
No. of pseudogenes (total)	212	399	214	173

a ncRNAs, noncoding RNAs.

The resulting genomes of the four isolates showed variation in size from 7.13 to 8.12 Mb. All assembled circular contigs possessed replication- and transfer-related genes, required for their independent existence as replicons. Their numbers varied from 6 to 9 in these isolates. Detailed statistics of final contigs are given in the table at https://doi.org/10.6084/m9.figshare.8970062.v1. The four strains also harbor multiple copies of ribosomal RNAs. Since rRNA genes were also present on three other contigs (p1, p2, and p4) in addition to the main chromosome, they are designated chromids (6), as described earlier for other members of the genus *Azospirillum* (7). The number of annotated genes also varied, from 7,491 to 6,518. Average nucleotide identity (ANI) values were measured using the ANI calculator of the Kostas server (8, 9). While ANI between MTCC4035 and MTCC4036 was 97.36%, it was only 93.65% between MTCC4038 and MTCC4039. The ANI of MTCC4035 or MTCC4036 with MTCC4038 or MTCC4039 was around 94%. Scans for bacteriophages and CRISPR arrays were performed using PHASTER (10) and PILER-CR v1.06 (11). A search for a CRISPR-Cas system was carried out using CRISPRCasFinder (12). The presence of bacteriophages was detected in these isolates (see https://doi.org/10.6084/m9.figshare.8970272.v1). CRISPR arrays were observed in MTCC4035 (*n* = 4) and MTCC4038 (*n* = 3), showing the presence of the complete class I type of CRISPR-Cas system. In MTCC4036 and MTCC4039, however, the discrete presence of CRISPR-Cas systems was also seen.

### Data availability.

Genome sequences of the strains MTCC4035, MTCC4036, MTCC4038, and MTCC4039 have been deposited in GenBank under the accession numbers CP032321 to CP032328, CP032330 to CP032338, CP032339 to CP032344, and CP032345 to CP032350, respectively. The genomes have been submitted under BioProject number PRJNA490424. The SRA accession numbers are SRR9046046 (MTCC4038), SRR9046049 (MTCC4036), SRR9046048 (MTCC4035), and SRR9046047 (MTCC4039). The versions described in this paper are the first versions.
